# First Documented Wild Population of the “Hunter Fly”, *Coenosia attenuata* Stein (Diptera: Muscidae) in North America

**DOI:** 10.3390/insects13110970

**Published:** 2022-10-22

**Authors:** Amos D. Kaldor, Joseph V. McHugh, Jason M. Schmidt, Xuelin Luo, Tara D. Gariepy, Brett R. Blaauw

**Affiliations:** 1 Department of Entomology, University of Georgia, Athens, GA 30606, USA; 2 Department of Entomology, University of Georgia, Tifton, GA 31793, USA; 3 Georgia Museum of Natural History, Athens, GA 30606, USA; 4 Experimental Statistics, University of Georgia, Tifton, GA 31793, USA; 5 London Research and Development Centre, Agriculture and Agri-Food Canada, London, ON N5V 4T3, Canada

**Keywords:** tiger fly, biological control, DNA barcoding, peach orchard, Southeast, phenology

## Abstract

**Simple Summary:**

The hunter fly, *Coenosia attenuata* Stein (1903) (Diptera: Muscidae), is a species of biological control significance typically found in greenhouses. Phylogenetic data suggests *Coenosia attenuata* originated in the Mediterranean region. Here, we provide the first report of a wild hunter fly population in North America. In 2020 and 2021, *Coenosia attenuata* was captured in pan traps set in Georgia and South Carolina peach orchards. Specimens collected across multiple sites over two years were identified with morphological keys and confirmed via DNA barcoding, providing strong evidence for an established population in the Southeastern USA.

**Abstract:**

*Coenosia attenuata* is a member of the *tigrina*-group of *Coenosia* (*sensu* Hennig 1964) and is a capable generalist predator in its larval and adult stages. *C. attenuata* is common in greenhouses worldwide, however, there are few documented cases of its presence in the wild. Here, we estimated *C. attenuata* presence in the southeastern USA peach orchards using pan traps. Over two years, a total of 717 specimens were collected from both commercially managed and fungicide-only managed peach orchards. *C. attenuata* is a known biological control agent in artificial greenhouse settings, but its impact on pest species in the wild is still unknown. For the first time in North America, we document an established wild population of *C. attenuata,* provide an overview of basic identification, and review potential benefits for biological control.

## 1. Introduction

The hunter fly, *Coenosia attenuata* Stein (1903) (Diptera: Muscidae), is a widespread predatory fly often associated with greenhouses [1]. Phylogenetic studies suggest that *C. attenuata* originated in the Mediterranean region, but is now known to occur in Europe, Asia, the Middle East, Africa, South America, and North America [1]. Across this wide distribution, the species exhibits limited mitochondrial genetic diversity, leading Seabra et al. [1] to propose that much of its current range is due to human-mediated expansion into greenhouses worldwide. Hoebeke et al. [2] hypothesized that the larvae are transported in potted plant soil. The larvae are soil-dwelling, polyphagous predators capable of living for 20–35 days even when prey is scarce [2]. 

*Coenosia attenuata* is a generalist predator in both adult and larval stages and, as such, is a biological control agent of multiple larval and adult greenhouse pests [3,4,5,6]. Common prey include: whiteflies (Hemiptera: Aleyrodidae), various Diptera, including fungus gnats (Sciaridae), leaf-miner flies (Agromyzidae), and pomace flies (Drosophilidae) [7,8]. The hunter fly thrives in artificial greenhouse environments and has been documented in New World greenhouses in Ecuador [9], Peru [9], Colombia [10], Costa Rica [11], Chile [12], Venezuela [13], Mexico [7], Honduras [14], Brazil [15], Uruguay [16], and the USA [17]. However, reports of *C. attenuata* outside of the greenhouse environment are uncommon, especially in the New World. In the first report of the species for North America, most records were greenhouse-based records, except for a single report of specimens captured in malaise traps in suburban Los Angeles, California [17]. The only other records of *C. attenuata* found in an open field setting in the New World are from South America, where adults were reported on baby’s breath flowers (Caryophyllaceae: *Gypsophila paniculata* L.) in Northeastern Brazil in 2016 and 2017 [15], and on blueberries (Ericaceae: *Vaccinium* sp.) in Chile [12].

**Figure 1:**
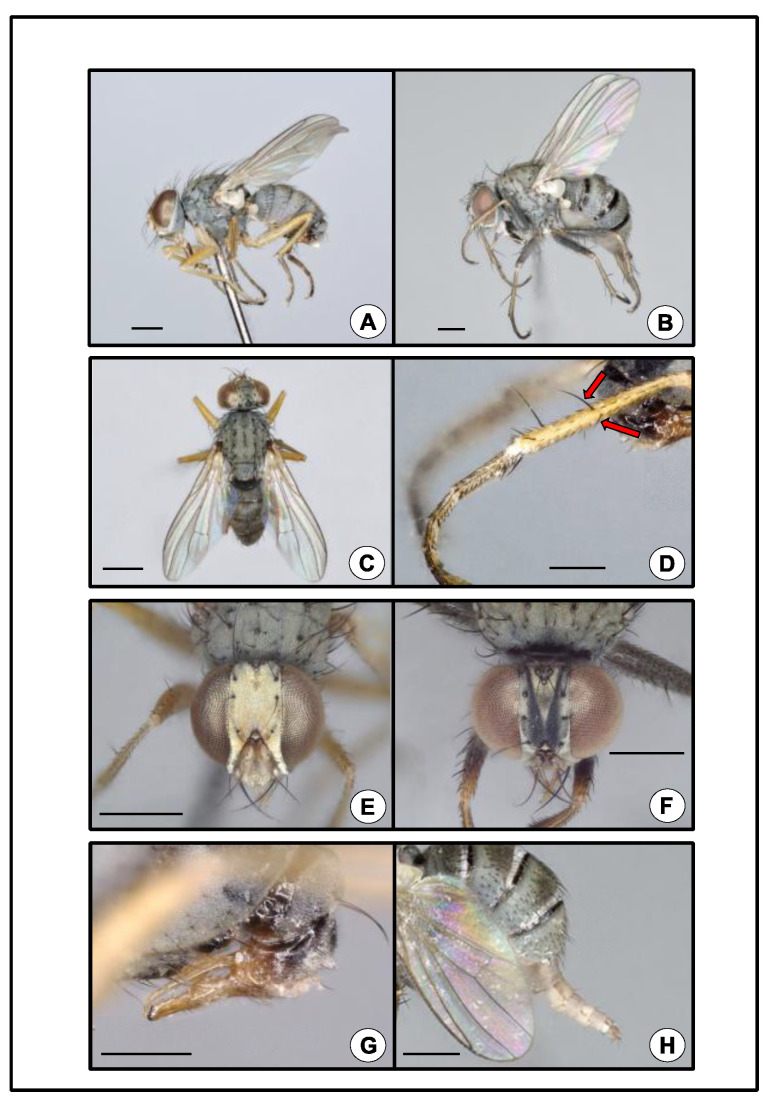
*Coenosia attenuata.* (**A**) Male, lateral. Scale bar = 0.5 mm. (**B**) Female, lateral. Note abdomen distended, exposing three black transverse stripes. Scale bar = 0.5 mm. (**C**) Male, habitus, dorsal. Scale bar = 0.5 mm. (**D**) Male, metathoracic tibia and tarsus, lateral. Arrows mark two major diverging setae at midlength. Scale bar = 0.25 mm. (**E**) Male, head, anterior. Scale bar = 0.5 mm (**F**) Female, head, anterior. Scale bar = 0.5 mm. (**G**) Male, abdominal terminalia, genitalia, lateral. Scale bar = 0.25 mm. (**H**) Female, abdominal terminalia, ovipositor, lateral. Scale bar = 0.5 mm.

During insect sampling in peach orchards in South Carolina (SC) and Georgia (GA), USA, in 2020 and 2021, 717 specimens of unknown fly species were collected in colored pan traps. The specimens were identified as *Coenosia attenuata* using morphological and molecular approaches (Figure 1). Here we document this new occurrence in peach orchard systems, provide a preliminary phenology for the species in peach orchards of the Southeastern USA, provide diagnostic information to assist in the recognition of this species based on the anatomy, and contribute COI barcodes to Genbank. 

## 2. Materials and Methods

### 2.1. Field Sites

**Figure 2:**
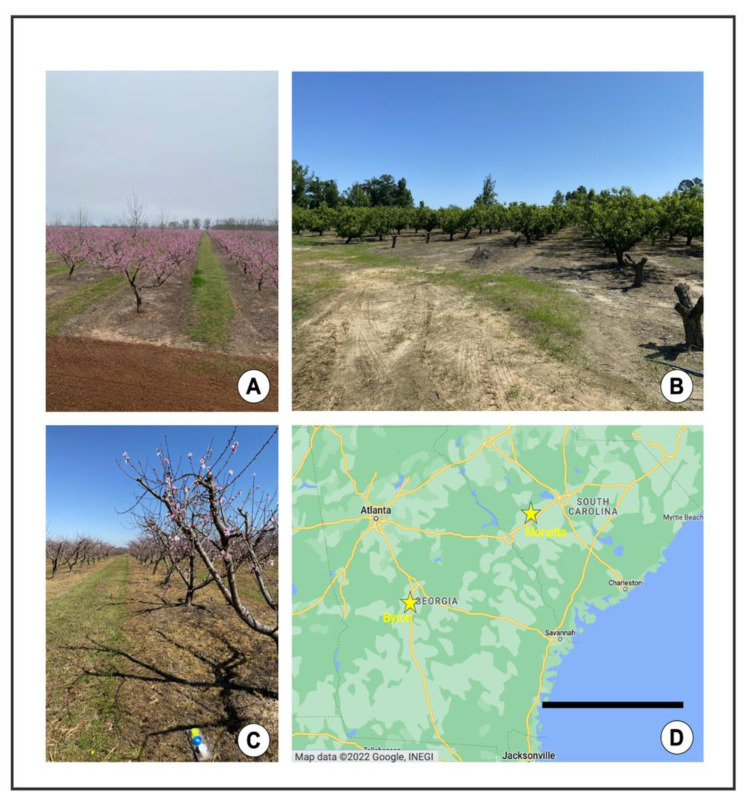
Field sites. (**A**) Commercial peach farm (high input), Byron, GA, 3/9/2021. (**B**) Organic peach farm (low input), Monetta, SC, 5/13/2021. (**C**) Commercial peach farm, Byron, GA, 3/16/2021. (**D**) Locations of field sites in Southeastern U.S. Map data © 2022 Google, INEGI (Google Maps) [20] Scale bar = 240 km.

The study was conducted in peach orchards in the Southeastern USA. The climate in the Southeastern USA is highly variable, experiencing extremely high temperatures in the summer and low temperatures in the winter months. There is also considerable humidity, and an average rainfall of between 44 and 52 inches per year [18]. The study sites were located in Byron, GA, and Monetta, SC, approximately 240 km apart (Figure 2). In GA, the six study sites were separated by 1–5 km, and in SC, the study sites were separated by 1–3 km. In an effort to maintain consistency, we selected orchards that were approximately the same size (1–3 ha). Peach orchards have largely sandy soil within the rows where peach trees are planted. The ground within and between rows is naturally well-lit, as trees are typically spaced 5 m apart, separated by grasses that are frequently mowed (Figure 2A–C). The orchards we sampled were managed with recommended practices following the 2022 Southeastern Peach Guide [19], with intensive commercial chemical applications or “high input” management, or “low input” (i.e., fungicide only in 2020, and organic fungicide and insecticide in 2021) management (Figure 2A–C). The high input orchards were treated primarily with a pyrethroid-based spray regimen, as well as supplementary fungicide sprays [19]. 

### 2.2. Sample Collection

We used pan traps colored with blue, white, or yellow fluorescent paint to estimate the activity of flower-visiting insects, such as hymenoptera, diptera, and lepidoptera [21,22]. Traps were constructed of plastic bowls (7.1 cm diameter) filled with dish soap (to break surface tension) and water. Twenty-seven traps were deployed per field site and were collected after 24 h. Traps were deployed in sets of three (blue, white, and yellow grouped together) and placed under nine trees in a transect pattern. Three trees along the edge of the orchard, three at 25 m towards the interior, and three at 50 m toward the interior. A total of 2244 traps were deployed over 17 sample dates. Traps were deployed monthly between July and October in 2020, and from March–September in 2021. During this study, many other insects were collected, however here we only report on *Coenosia attenuata.*

### 2.3. Sample Processing

While sorting the pan traps, we discovered an unusual dipteran, and initially we sent representative specimens to E. Richard Hoebeke (University of Georgia Collection of Arthropods) and Adrian C. Pont (Oxford University) for identification. Hoebeke and Pont confirmed the identification of the fly as *Coenosia attenuata*. Remaining traps were then sorted to document all specimens of this fly, and subsequent specimens were morphologically identified by the authors using Hoebeke et al.’s guide [2]. *Coenosia attenuata* is a member of the *tigrina*-group of *Coenosia* (*sensu* Hennig 1964) [23], which includes Old World species that can be diagnosed by the occurrence of two major diverging bristles near the midlength of the anterior and anterodorsal surfaces of the hind tibia (Figure 1D). Two other species of this group have been introduced into North America, *Coenosia tigrina* F. (1775) and *Coenosia humilis* Meigen (1826) [2]. *Coenosia attenuata* can be distinguished from these two species based on its smaller size, having a body length of 2.5–3.0 mm for males and 3.0–4.0 mm for females, (*C*. *tigrina*: 4.75–5.75 mm, male and 5.75–7.0 mm, female; *C. humilis*: 3.0–3.5 mm, male and 3.0–5.5 mm, female), and by the yellow color of the femora in males (*C. tigrina* femora are black with a reddish-yellow apex; *C. humilis* femora are black) [2]. In addition, the form of the male genitalia of *C. attenuata* is diagnostic (Figure 1G). A detailed taxonomic description and natural history summary for *C. attenuata* are given by Hoebeke et al. [2].

Three male specimens were identified using DNA barcoding of the cytochrome oxidase 1 (CO1) gene. Genomic DNA was extracted from individual flies (whole body extraction) using the Qiagen DNeasy Blood and Tissue kit, following the manufacturer’s protocol. Extracted DNA was stored at −20 °C. A negative extraction control that contained all Qiagen buffers for extraction and Proteinase K solutions was included. The DNA barcode region of the CO1 gene was amplified using standard DNA barcoding primers [24], following the protocol described by Cutler et al. [25]. PCR products were sent for purification and sequencing at Eurofins (©Eurofins Scientific 2021). Purified PCR products were sequenced bidirectionally. Using Codon Code Aligner version 9.0.1, forward and reverse sequences were assembled, aligned, and edited to trim the remaining primer sequences. Prepared sequences were then “blasted” against all sequence records in public databases of the Barcode of Life Data System (BOLD) and the National Center for Biotechnology Information (NCBI).

A representative male and female were imaged by transferring preserved specimens with 75% ethanol to amyl acetate for 48 h before being mounted on a minute pin and air dried. Specimens were photographed using the automated image rendering of a Keyence VHX-7000 digital imaging microscope (Keyence, Itasca, IL, USA). Voucher specimens were deposited in the University of Georgia Collection of Arthropods (UGCA) in Athens, GA, USA. 

### 2.4. Statistical Analyses

We fitted several statistical models to test if hunter-fly counts were influenced by sampling date, trap color, or management intensity (i.e., high or low chemical input), and to assess male:female sex ratios. In each of the models, we fitted general mixed effect models (GLMMs) using the GLIMMIX procedure. AR1 covariance structure of errors was used in testing for seasonal variation, which considered repeated measures. To test if hunter fly counts were influenced by pan trap color, we fitted a GLMM of hunter fly pooled counts by field. Trap color was the fixed effect, and transect and date were set as random effects. To ask whether hunter flies in the Southeast are multivoltine, we assessed significant seasonal variation in hunter fly counts for each year using GLMMs with date as the fixed effect and transects nested in fields used as random effects. Lastly, management intensity (i.e., high or low chemical input) in orchards may affect the abundance of hunter flies. Therefore, in similar structured models, we analyzed the effect of management and field position (i.e., transect) on hunter fly counts using GLMMs with management intensity, transect, and the interaction between management intensity and transect as fixed effect terms with date nested within field as random effects. The differences in the model structure of fixed effects and random effects were needed to test different hypotheses. In all cases, natural log transformed hunter fly counts were used as the response variable, which improved normality and homogeneity of variance. The statistical analyses were conducted in SAS version [9.4] (2013) by SAS Institute Inc., Cary, NC, USA. 

## 3. Results

*Coenosia attenuata* is a small, light gray fly with a muscoid body form (Figure 1A–C). The tarsi are long with black setulae and at rest are held slightly curved, giving a raptorial appearance (Figure 1A,B,D). The sexes are dimorphic. Males are smaller than females (see above). The male head has a bright silvery-white pruinose vertex, frons, parafacials, and lunule (Figure 1E). The female head is pruinose and gray with two dark converging longitudinal stripes on the frons (Figure 1F). The legs are yellowish in males with slightly darker tarsi (Figure 1A,D). The legs of females are grayish, especially on the femora (Figure 1B). The abdomen is uniformly light gray in males (Figure 1A,C,G) or has some indistinct darker blotches, but in females, the abdomen bears three distinct black transverse stripes (Figure 1B,H). The morphological characteristics described above, and the COI sequence analysis confirmed the collected specimens to be *C. attenuata*. All three individuals that were barcoded yielded a 658-bp fragment (Genbank accession numbers: ON257860–ON257862), and were consistent (i.e., 100% overlap and >99% identity match) with public sequences for *C. attenuata* on both BOLD and Genbank.

**Figure 3:**
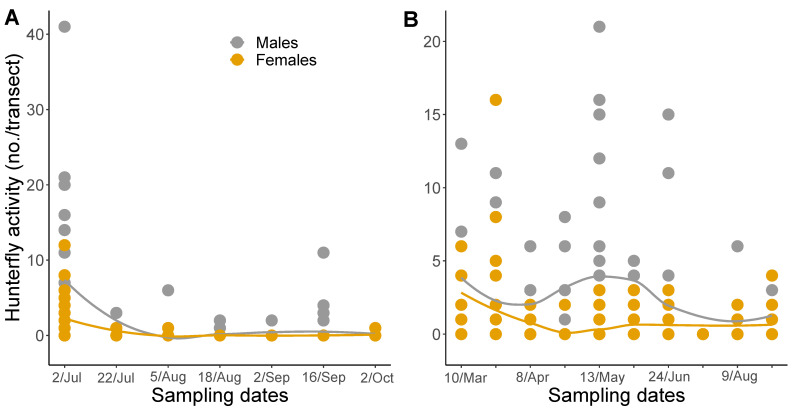
Seasonal summary of mean counts of *Coenosia attenuata* observed in pan traps combined by transect for (**A**) 2020 and (**B**) 2021. Line color indicates: Male = gray, Female = gamboge. Data points represent the mean sum total of hunter flies captured in blue, white, and yellow pan traps for a total of nine traps per field. The solid lines are loess smoothing curves with corresponding shaded 95% CI to display the estimated fluctuation in captures over the season.

Overall, we captured 609 individuals in GA and 108 in SC for a total of 717 *C. attenuata* over the two-year study. In 2020, we captured a total of 231 and in 2021, 486 C. *attenuata*. For both male and female hunter flies, in 2020 there was significant variation in counts throughout the season (F_6,30_ = 5.57, *p* = 0.0006; F_6,30_ = 8.67, *p* ≤ 0.0001, respectively), with a peak in July (Figure 3A). In 2020, there were significantly more flies caught on July 2nd than on all other sample dates, expect for another peak in capture on September 16th (Tukey-Kramer). There was marginally significant variation in seasonal data for male and female hunter flies in 2021 (F_9,34_ = 1.95, *p* = 0.0783; F_9,34_ = 1.98, *p* = 0.0734, respectively; Figure 3B).

**Figure 4:**
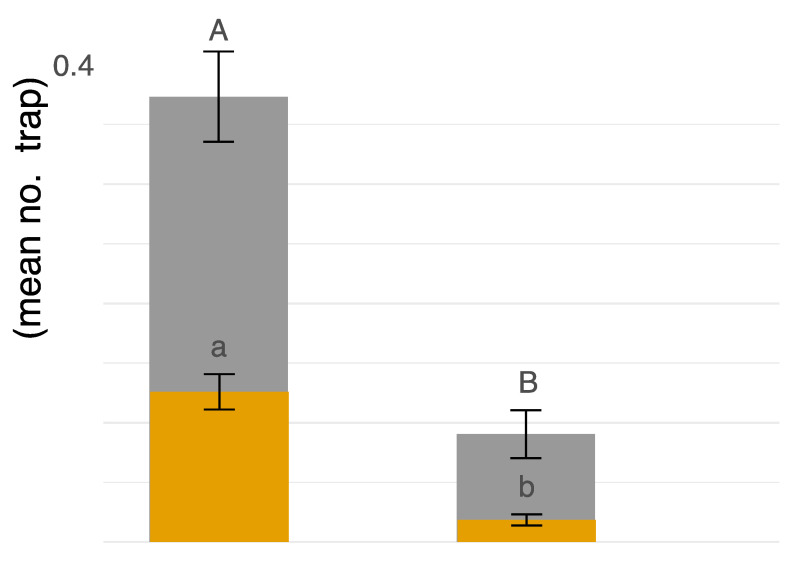
Male = grey, Female = gamboge. On the x-axis, “high” is high-input commercial chemical management, “low” is low-input fungicide-only chemical management. On the y-axis, blue, white, and yellow traps were pooled together, and displayed as average count of flies per trap location. The uppercase letters represent linear contrasts for males and lowercase letters for females in relation to management strategy (α = 0.05).

*Coenosia attenuata* was found at both high-input and low-input managed peach orchards during both years of the study. Our sampling efforts over two years produced a greater number of specimens from the high input sites (n = 595) than the low input sites (n = 122) (Figure 4). Trap color appears to be a significant predictor of hunter-fly counts observed in traps for both males (F_2,255_ = 34.78, *p* ≤ 0.0001) and females (F_2,255_ = 16.47, *p* ≤ 0.0001). A greater number of hunter flies were observed in white bowls (n = 585) as compared to either blue (n = 45) or yellow (n = 77) (t = −7.65, df = 255, *p* ≤ 0.0001; t = 6.70, df = 255, *p* ≤ 0.0001, respectively).

The number of male hunter flies captured was influenced by management strategy (F_1,179_ = 16.64, *p* ≤ 0.0001; Figure 4), no significant influence of field transects (F_2,179_ = 2.50, *p* = 0.0847), and no interaction between management and field transects (F_2,179_ = 1.49, *p* = 0.2272) (Figure 4). More males were observed in high input management than in low input (t = 4.08, *p* ≤ 0.0001) (Figure 4). For females, management strategy had a significant effect on the number of flies captured (F_1,179_ = 13.61, *p* ≤ 0.0001), with no transect effect (F_2,179_ = 0.98, *p* = 0.3773) or interaction between management and field transect (F_2,179_ = 1.12, *p* = 0.3301). The management effect is explained by higher numbers of female hunter flies observed in the high input system (t = 5.29, *p* ≤ 0.0001) (Figure 4). Lastly, abundance patterns showed male hunter flies were more commonly captured, providing evidence of male biased sex ratios with on average 3.145 males per female (X^2^ = 191.98, *p* < 0.0001) (Figure 4).

## 4. Discussion

Here, we report the first wild population of *C. attenuata,* the hunter fly, in the peach agroecosystems of Georgia and South Carolina, USA. The number of flies captured varied according to management, sex ratio, as well as trap color. Overall, *C. attenuata* was present in high-input, commercially managed orchards, USDA organically certified orchards, and in low-input, fungicide-only managed research orchards. In Turkey, *C. attenuata* was monitored in a greenhouse that underwent chemical treatments to combat whiteflies. All four chemical treatments of insecticides and fungicides appeared to have low-to-no impact on hunter fly populations [4]. This suggests that *C. attenuata* may be tolerant of some insecticide and fungicide applications, demonstrating their potential value as a biological control agent in commercially managed field settings. 

As a predator, adult hunter flies rely primarily on visual cues. Their hunting behavior involves perching in well-lit areas, waiting for flying insects to venture nearby, darting out to capture the prey in the air, and returning to their perch [26,27]. This precise mid-air attack is called “hawking” hunting behavior, and is a strategy shared with other insects, such as Anisoptera (Odonata) and Asilidae (Diptera) [26]. Hunter flies can attack prey from any angle (launching from the ceiling, a wall, or the ground), minimizing their flight time and efficiently expending energy. Adults kill their prey by stabbing the cervix (neck) area with their proboscis, sometimes partially decapitating their prey, before returning to their perch to drink the nutrients [28]. Hunter flies were recorded attacking flying insects near their perch without attempting to eat them, exhibiting almost territorial behavior [29]. Interestingly, Mateus et al. [28] found that an attack was not provoked if prey walked near *C. attenuata;* only a nearby flight would trigger an attack. This provoked hunting behavior of adults was observed again in laboratory feeding experiments with different prey species. In a controlled laboratory experiment, hunter fly adults preyed most heavily on adult fungus gnats, followed by adult shore flies and adult whiteflies [30,31]. Whiteflies are mostly sedentary when feeding, so it would follow that they are attacked less often by the hunter flies [28]. We did not monitor the feeding habits of the *C. attenuata* in this study. However, many of the known prey taxa of *C. attenuata* were present in our pan trap bycatch. It is possible that *C. attenuata* are drawn to the prey taxa attracted to the pan traps, using these pseudo “flowers” as a hunting site [15,32]. The feeding habits and biological control contributions of *C. attenuata* in the southeastern USA’s peach orchards should be considered in future studies. 

The number of hunter flies captured in peach orchards varied seasonally. Capture data suggest anecdotal peaks in activity during early April, late May, and early July (Figure 3). Under laboratory conditions, the lifecycle of *C. attenuata* takes 26 days to complete [33]. The only record of a wild population of *C. attenuata* phenology suggests the fly is multivoltine in Turkish cotton fields [34]. Wild populations of a closely related species, *Coenosia tigrina*, in Michigan onion fields were also found to be multivoltine [35]. Similarly, species of another hunter-fly genus, *Limnophora* Robineau-Desvoidy (1830) (Muscidae: Coenosiinae), were found to be multivoltine in tufa barriers in Croatia [36]. However, our current data do not suggest multiple statistically significant peaks in counts throughout 2020 and 2021, so we cannot conclude if *C. attenuata* are multivoltine in the southeastern part of the USA.

The sex ratio of reported male and female hunter flies differs between a greenhouse, laboratory, and field settings as well as according to sample methodology. In this study, the sex ratio of hunter flies was male-biased; 3.145 Male:Female (Figure 4), while greenhouse systems appeared to be female-biased; 0.25 M:F ratio [28]. Furthermore, under optimized laboratory-rearing conditions, emerging flies had a sex ratio of 0.67 and 0.69 M:F [32,37]. With the number of confounding variables between studies, it is difficult to speculate on what caused the different sex ratios. Environmental conditions may have affected the sex ratio (e.g., the moist soil in greenhouses, or coconut fiber/black peat mixture for rearing, versus the dry sandy substrate of peach farms). Alternatively, sampling methodology may affect sex-specific capture rates (e.g., suction sampling, sticky cards, and pan traps). In addition, we found that both male and female hunter flies were most abundant in the white-colored pan traps. However, factors that contribute to different sex ratios of *C. attenuata* should be explored in future studies.

## 5. Conclusions

At this time, we cannot predict the impact of the *Coenosia attenuata* establishment in the southeastern part of the USA’s agroecosystems. However, based on hunter-fly biology and life history traits, this species should be considered a potential biological control agent. As such, future studies in the region should be mindful of this potentially significant addition to the local insect fauna. 

## Data Availability

The data presented in this study are available on request from the corresponding author.
